# Supplementary material for: Structure and Properties of a Metallocene Polypropylene Resin with Low Melting Temperature for Melt Spinning Fiber Application
Source: Polymers (Basel). 2019 Apr 22;11(4):729. doi: 10.3390/polym11040729 (PMC6523879; doi:10.3390/polym11040729)
Supplement: Supplementary file 1 [file polymers-11-00729-s001.pdf]

Supporting Information

Structure and Properties of a Metallocene Polypropylene Resin with Low Melting Temperature for Melt Spinning Fiber Application

Renwei Xu[1], Peng Zhang[1], Hai Wang[1,*], Xu Chen[1], Jie Xiong[2], Jinpeng Su[3], Peng Chen[3, 4,*], Zhicheng Zhang[2,*]

[1] Lanzhou Petrochemical Research Center, Petrochina, Lanzhou, China, 730060. xurenwei@petrochina.com.cn (R. X.); zhangpeng931@petrochina.com.cn (P. Z.); chenxu001@petrochina.com.cn (X. C.).

[2] Xi'an Key Laboratory of Sustainable Energy Materials Chemistry; Department of Applied Chemistry; School of Science; Xi'an Jiaotong University; Xi'an, P. R. China, 710049. xiongjiely@stu.xjtu.edu.cn (J. X.).

[3] Ningbo Key Laboratory of Polymer Materials, Ningbo Institute of Materials Technology and Engineering (NIMTE), CAS, Ningbo, China, 315201. sujinpeng@nimte.ac.cn (J. S.).

[4] Center of Materials Science and Optoelectronics Engineering, University of Chinese Academy of Sciences, Beijing, China, 100049.

***** Correspondence: wanghai2@petrochina.com.cn; pchen@nimte.ac.cn; zhichengzhang@mail.xjtu.edu.cn; Tel.: +86-29-82668546.

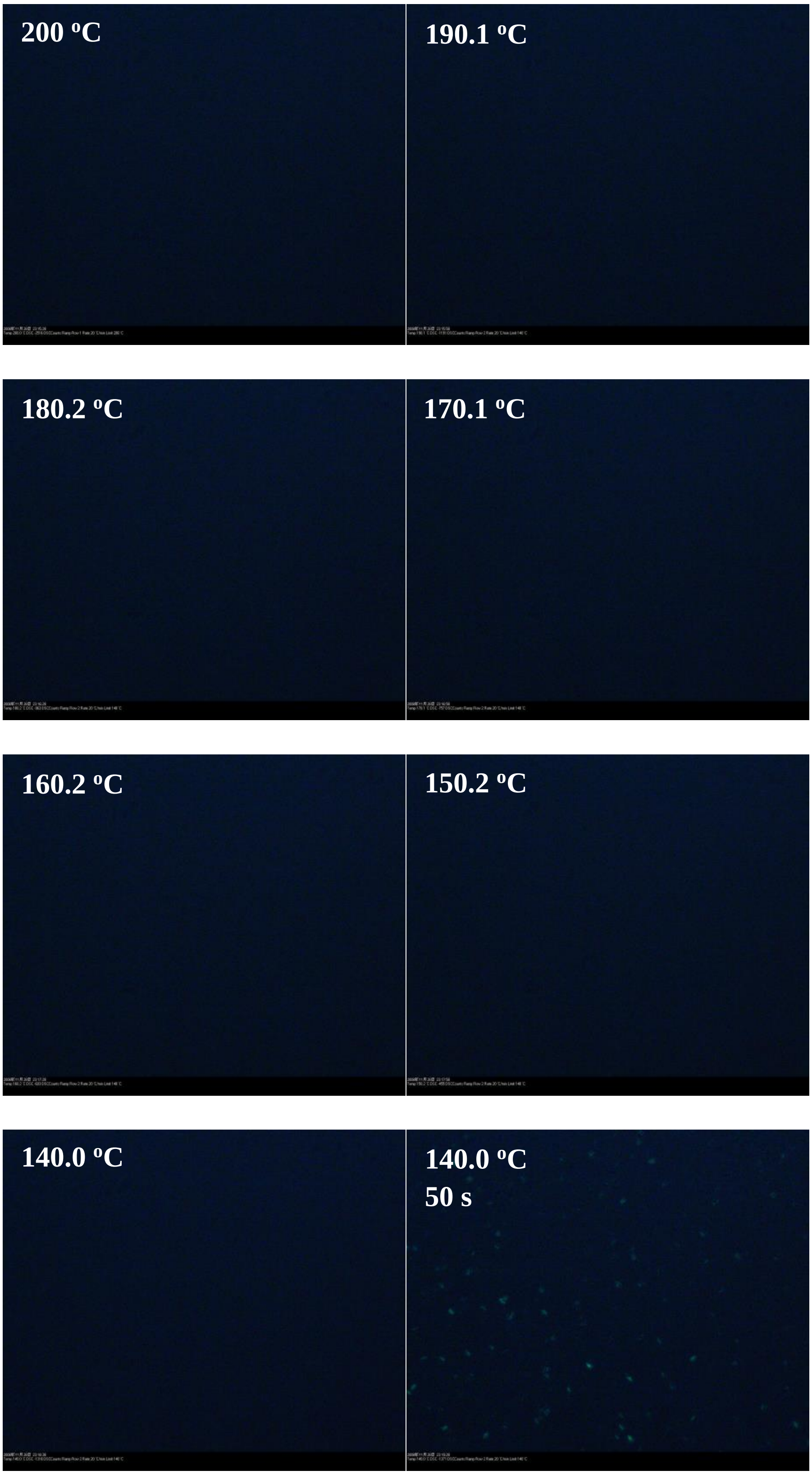
200 ºC
190.1 ºC
180.2 ºC
170.1 ºC
160.2 ºC
150.2 ºC
140.0 ºC
140.0 ºC
50 s

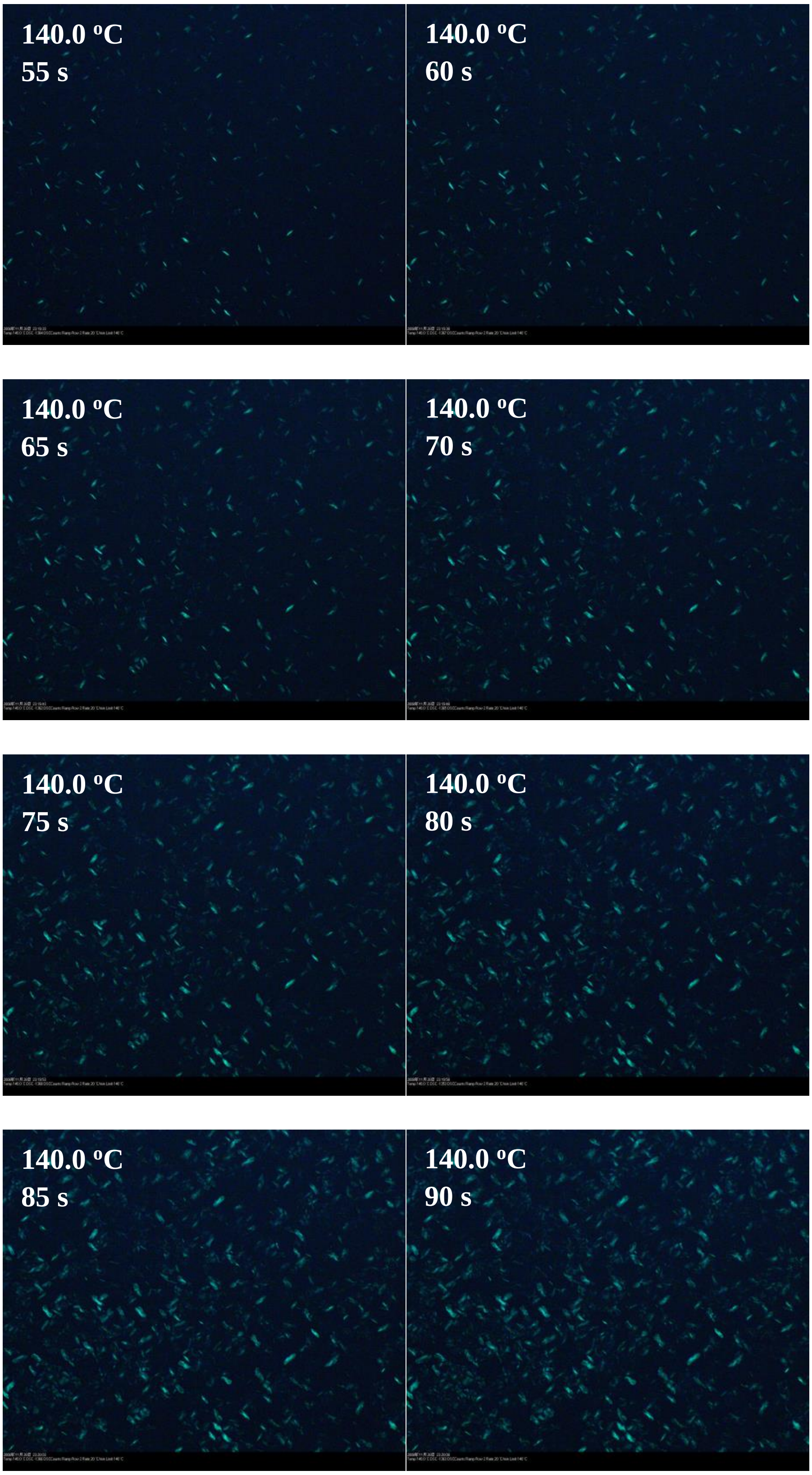
140.0 ºC
55 s
140.0 ºC
60 s
140.0 ºC
65 s
140.0 ºC
70 s
140.0 ºC
75 s
140.0 ºC
80 s
140.0 ºC
85 s
140.0 ºC
90 s

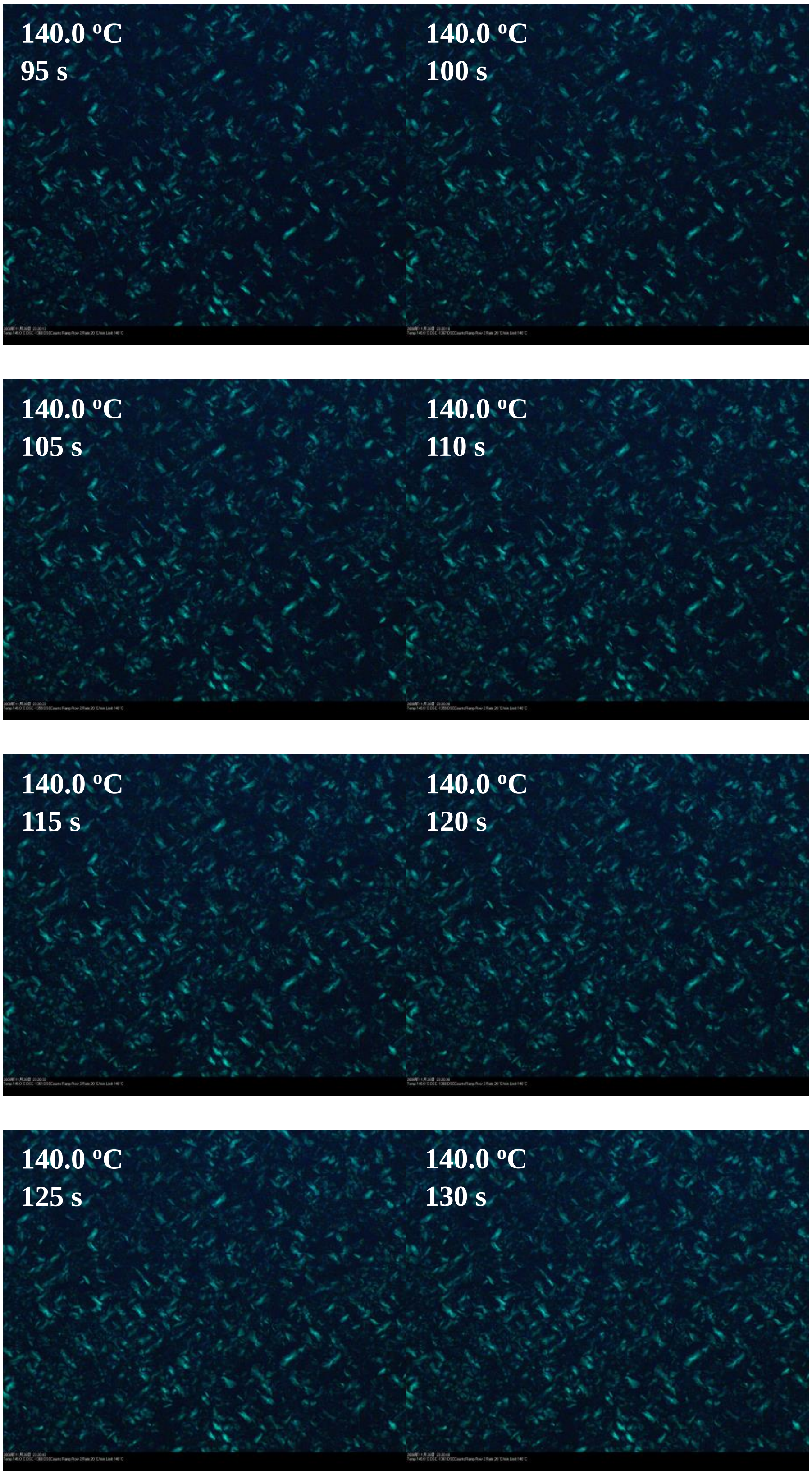
140.0 ºC
95 s
140.0 ºC
100 s
140.0 ºC
105 s
140.0 ºC
110 s
140.0 ºC
115 s
140.0 ºC
120 s
140.0 ºC
125 s
140.0 ºC
130 s

iPP-1

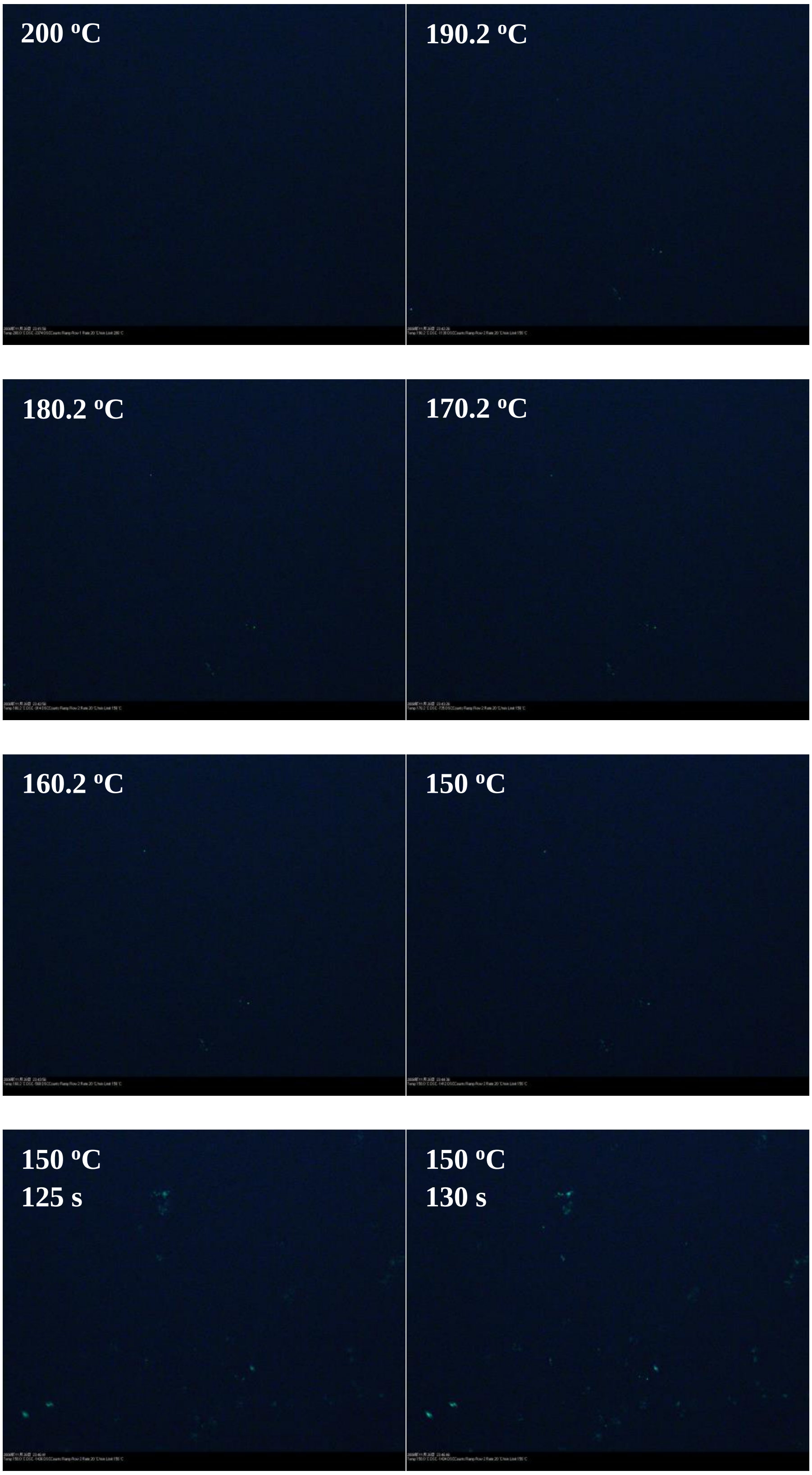
200 ºC
190.2 ºC
180.2 ºC
170.2 ºC
160.2 ºC
150 ºC
150 ºC
125 s
150 ºC
130 s

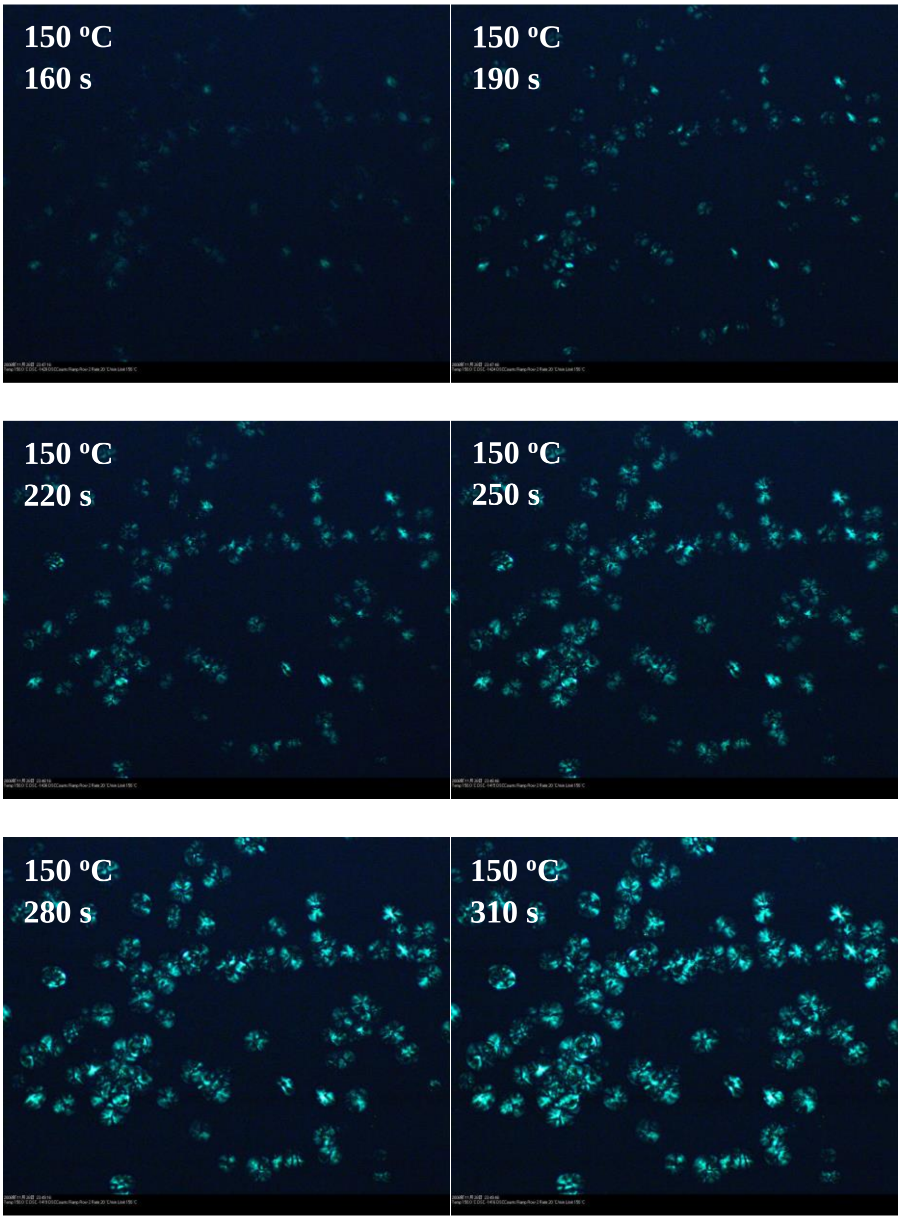
150 ºC
160 s
150 ºC
190 s
150 ºC
220 s
150 ºC
250 s
150 ºC
280 s
150 ºC
310 s

iPP-2

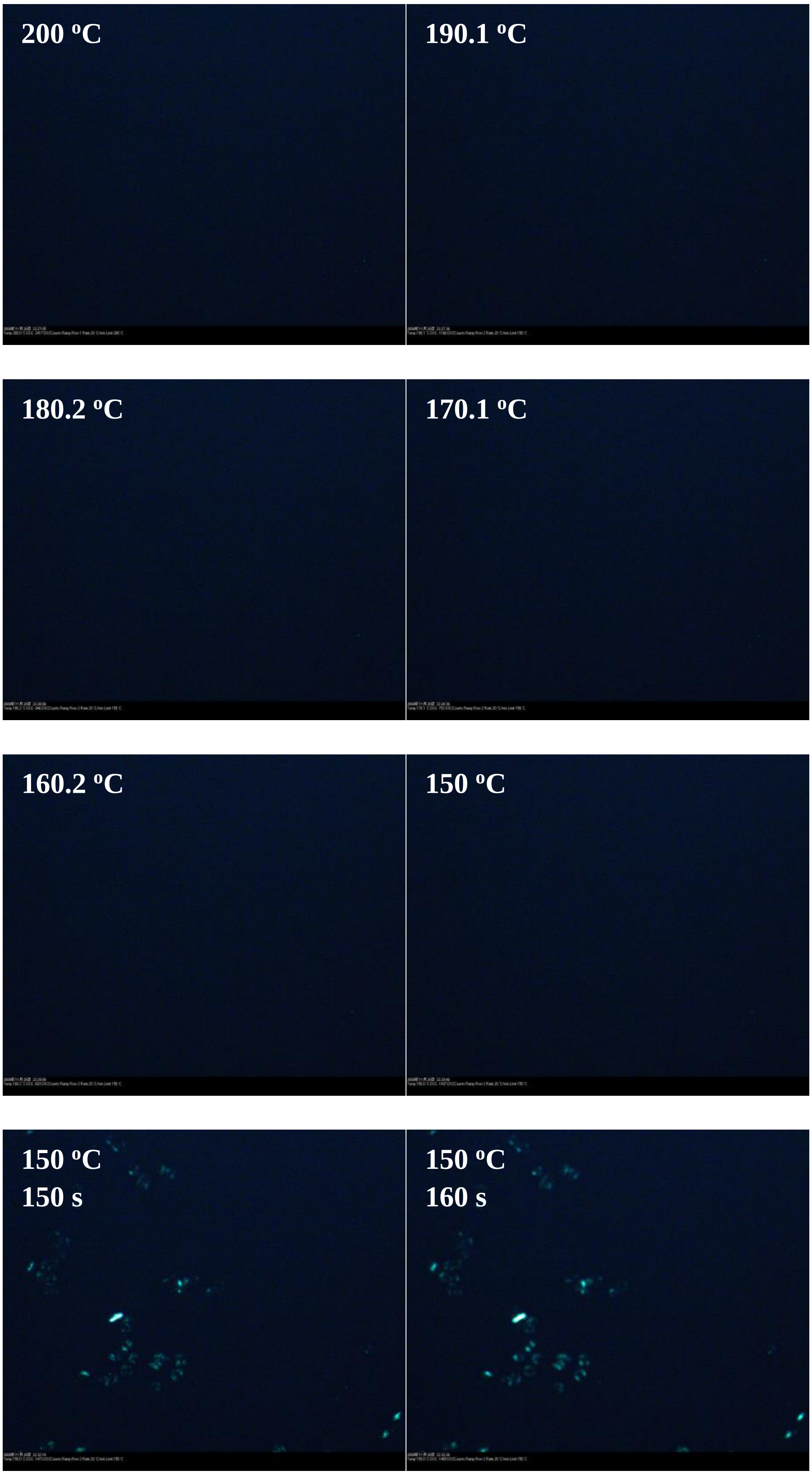
200 ºC
190.1 ºC
180.2 ºC
170.1 ºC
160.2 ºC
150 ºC
150 ºC
150 s
150 ºC
160 s

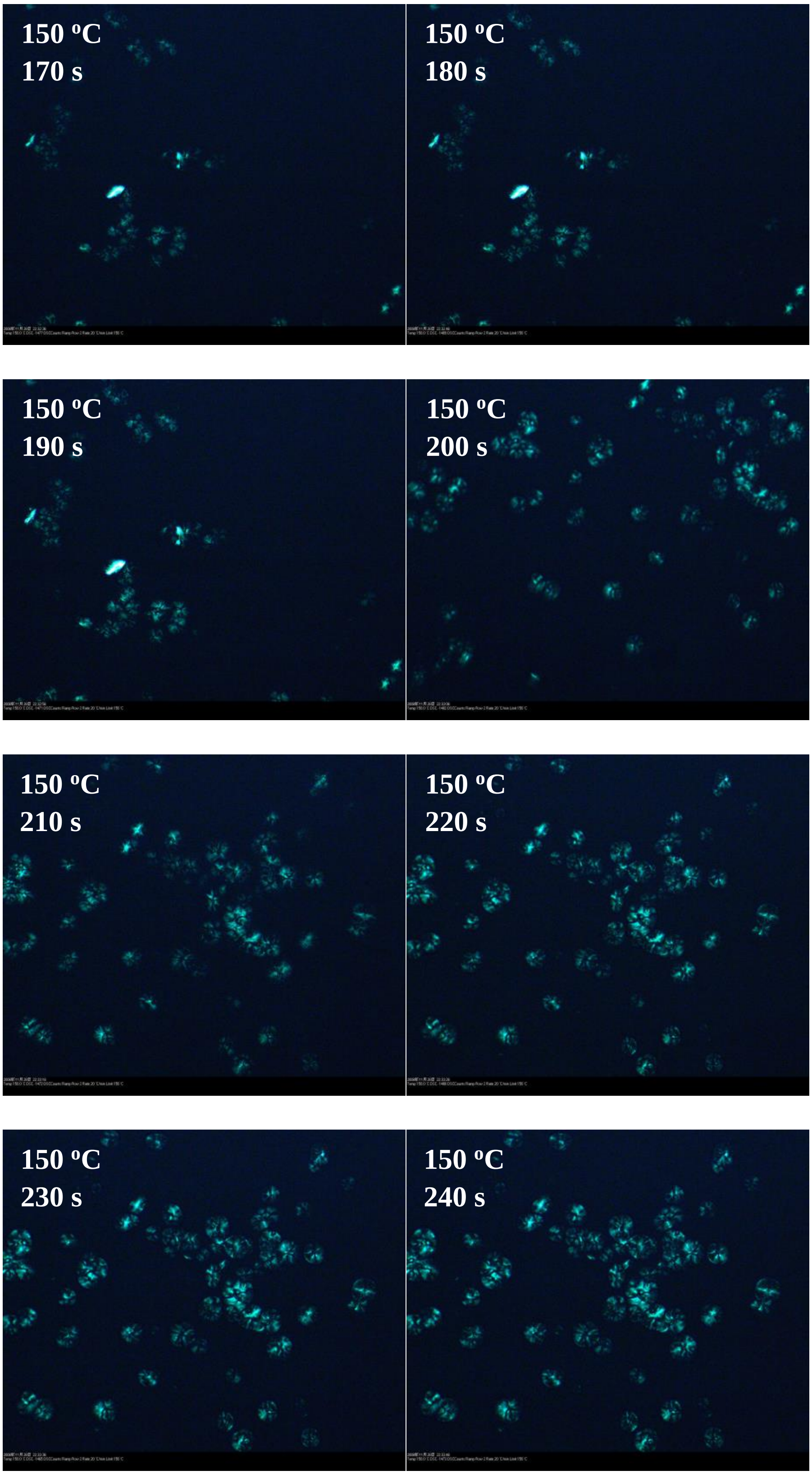

150 ºC
170 s
150 ºC
180 s
150 ºC
190 s
150 ºC
200 s
150 ºC
210 s
150 ºC
220 s
150 ºC
230 s
150 ºC
240 s

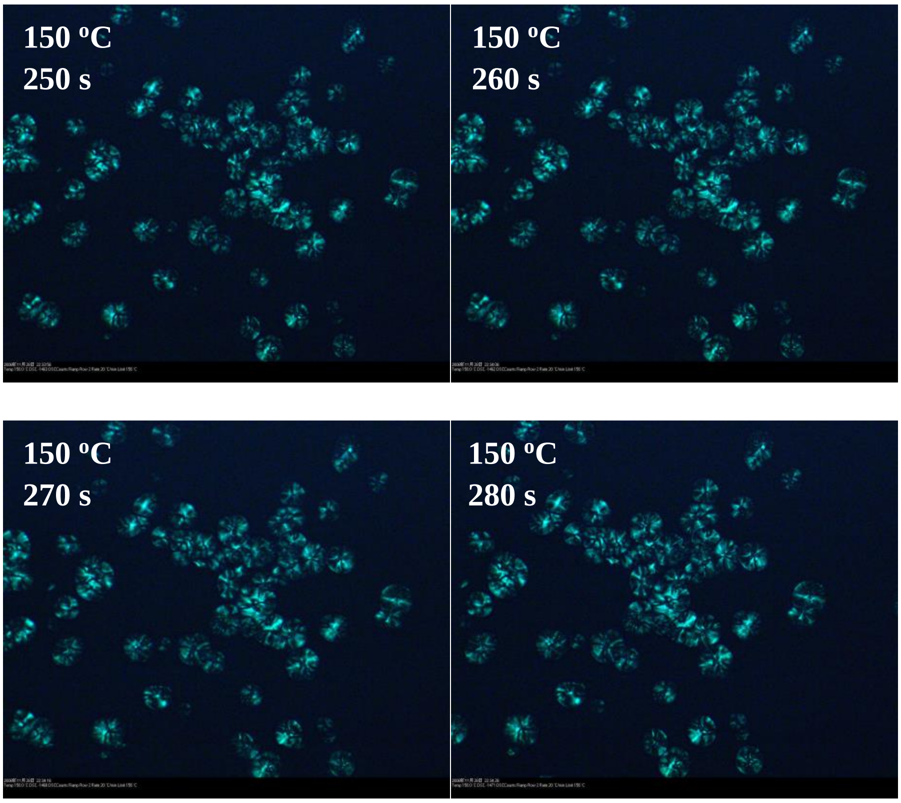

iPP-3

Figure S1. Morphologies of the iPP samples under a polarizing microscope.

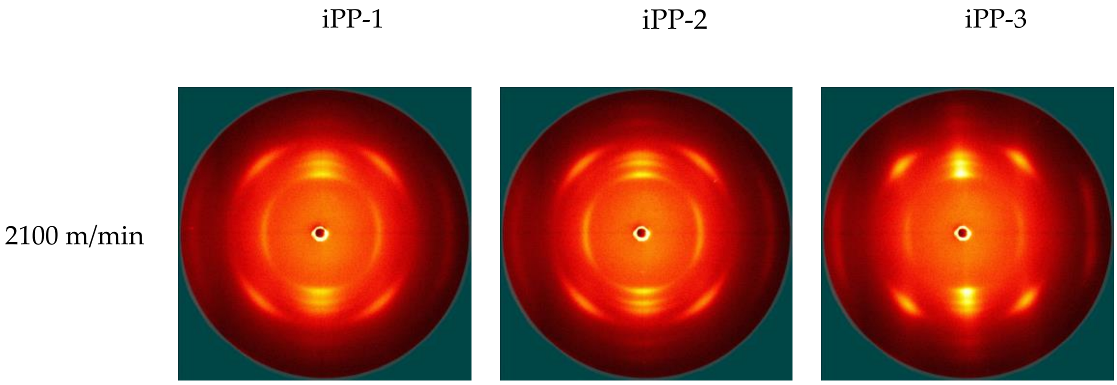

Figure S2. 2D WAXD patterns of as-spun iPP fibers.

iPP-1 iPP-2 iPP-3

2100 m/min

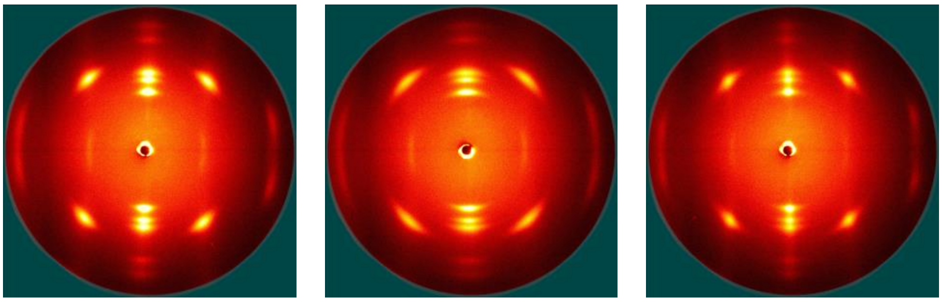

Figure S3. 2D WAXD patterns of drawn iPP fibers (draw ratio: 1.6).

iPP-1 iPP-2 iPP-3

2100 m/min

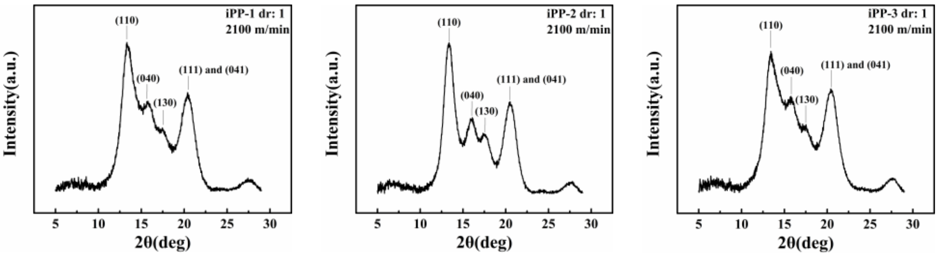

Figure S4. 1D WAXD curves of as-spun iPP fibers.

iPP-1 iPP-2 iPP-3

2100 m/min

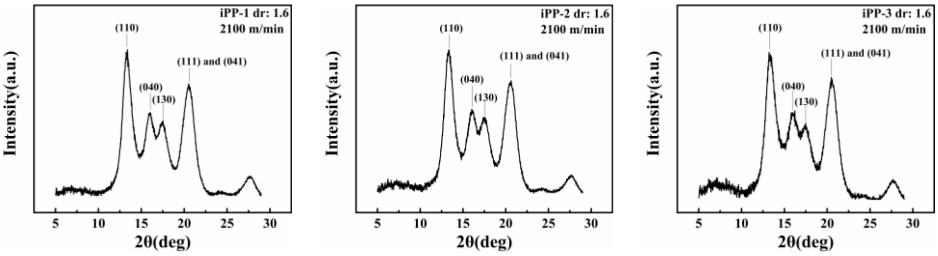

Figure S5. 1D WAXD curves of drawn iPP fibers (draw ratio: 1.6).
